# Combining the Finite Element Method with Structural Connectome-based Analysis for Modeling Neurotrauma: Connectome Neurotrauma Mechanics

**DOI:** 10.1371/journal.pcbi.1002619

**Published:** 2012-08-16

**Authors:** Reuben H. Kraft, Phillip Justin Mckee, Amy M. Dagro, Scott T. Grafton

**Affiliations:** 1 Soldier Protection Sciences Branch, Protection Division, U.S. Army Research Laboratory, Aberdeen Proving Ground, Maryland, United States of America; 2 Dynamic Science, Inc., Aberdeen Proving Ground, Maryland, United States of America; 3 Department of Psychology, University of California, Santa Barbara, Santa Barbara, California, United States of America; Indiana University, United States of America

## Abstract

This article presents the integration of brain injury biomechanics and graph theoretical analysis of neuronal connections, or connectomics, to form a neurocomputational model that captures spatiotemporal characteristics of trauma. We relate localized mechanical brain damage predicted from biofidelic finite element simulations of the human head subjected to impact with degradation in the structural connectome for a single individual. The finite element model incorporates various length scales into the full head simulations by including anisotropic constitutive laws informed by diffusion tensor imaging. Coupling between the finite element analysis and network-based tools is established through experimentally-based cellular injury thresholds for white matter regions. Once edges are degraded, graph theoretical measures are computed on the “damaged” network. For a frontal impact, the simulations predict that the temporal and occipital regions undergo the most axonal strain and strain rate at short times (less than 24 hrs), which leads to cellular death initiation, which results in damage that shows dependence on angle of impact and underlying microstructure of brain tissue. The monotonic cellular death relationships predict a spatiotemporal change of structural damage. Interestingly, at 96 hrs post-impact, computations predict no network nodes were completely disconnected from the network, despite significant damage to network edges. At early times (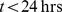) network measures of global and local efficiency were degraded little; however, as time increased to 96 hrs the network properties were significantly reduced. In the future, this computational framework could help inform functional networks from physics-based structural brain biomechanics to obtain not only a biomechanics-based understanding of injury, but also neurophysiological insight.

## Author Summary

According to the Centers for Disease Control and Prevention in the United States, approximately 1.7 million people, on average, sustain a traumatic brain injury annually. During the last few decades, brain neurotrauma biomechanics has been an active area of research involving medical clinicians and a broad range of scientists and engineers. In addition, advances and fast growth of human connectomics continues to reveal new insights into the damaged brain. With recent advances in computational methods and high performance computing, we see the need and the exciting possibility to merge brain neurotrauma biomechanics and human connectomics science to form a new area of investigation - connectome neurotrauma mechanics. For neurotrauma, the idea is simple - inform human structural connectome analysis using physics-based predictions of biomechanical brain injury. If successful, this technique may be further used to inform human functional connectome analysis, thus providing a new tool to help understand the pathophysiology of mild traumatic brain injury.

## Introduction

The finite element method is often used to study neurotrauma [1]–[9] and continues to emerge as a useful tool in the field of neuroscience [10]–[13]. Models continue to advance in biofidelity by incorporating an increased level of anatomic detail [4], [13], [14], improved representation of the material behavior at various loading rates [15]–[20], and advanced measures and predictions of injury [8], [21], [22]. Finite element models are commonly used to understand the biomechanics of brain and skull deformation when the head is subjected to insult, leading to improved insight into mechanisms of acute injury. For example, modeling axonal injury mechanisms within white matter of the brain has been the focus of some recent efforts and provides a means to relate an insult to a cellular injury mechanism [8]. By using diffusion tensor imaging (DTI) fiber tractography, the structural orientation of neuronal axonal bundles can be incorporated into the finite element model and can be used to compute the axonal strain during brain white matter deformation [8], [23]. Then, by using an axonal injury threshold, the occurrence of diffuse axonal injury (DAI) is predicted [8]. Wright and Ramesh [8] show that the degree of injury predicted is highly dependent on the incorporation of the axonal orientation information and the inclusion of material anisotropy into the constitutive model for white matter. By modeling the underlying mechanism of DAI, an enhanced understanding of the neurotrauma is attained through a spatiotemporal description of tissue deformation. In general, finite element simulations may help to elucidate the injury mechanisms of neurotrauma.

As finite element models advance, experimentally based models of neurotrauma also continue to become more sophisticated, ranging from the macroscopic [24], [25] to the cellular level [26]–[29]. Various biomechanical and physiological injury thresholds for neurotrauma have been proposed in the past, including intracranial pressure [21] and strain [8], [30], [31]. While these thresholds offer an immediate prediction of injury, they lack a long-term description of functional degradation. A time-evolving injury model is attractive since the biological response occurs on a slower time scale than an injurious stimulus as a consequence of mechanotransduction cascades [27]. There have been significant efforts to develop empirically based time-evolving cellular injury thresholds, which many times use in vitro cellular and tissue culture models. Morrison III et al. [27] suggest that for brain biomechanics, neuronal culture models that accurately mimic specific brain features can be used to explore tissue properties and tolerances or thresholds to mechanical loading. Cell death has been primarily used as a definition of injury within the neuronal culture model community and has been applied to determine tissue-level tolerance criteria using local values of axonal strain, strain rate, and time from “insult” [32], [33]. Furthermore, as Morrison III et al. [27] point out, empirical functions for cellular death based on culture models could be incorporated into finite element analyses, thereby enabling biological predictions to supplement mechanical predictions of local tissue stress and strain. In the study presented here, this concept is further explored and used as a bridge to a network-based analysis of the brain.

As physics-based models become more capable of predicting tissue-level injury mechanisms from improved computational and experimental resources for biomechanics, there remains a need to understand how structural damage in a given location of the brain evolves, and how it may influence functional or cognitive performance over time. Such a goal is complex and difficult. For example, as Kaiser et al. point out [34], in some instances the brain can be robust to physical damage, and in other instances physical damage can cause severe functional deficits. Nevertheless, Kaiser et al. [34] pursue the important and unanswered question: Are the severity and nature of the effects of localized damage predictable? To help explore the answer to this question, tools are being developed for the quantitative analysis of brain network organization, based largely on graph theory [35], [36]. Typically, network nodes represent brain regions, often obtained from high resolution magnetic resonance imaging (MRI). The network links or edges between brain regions represent interregional pathways that convey neuronal signals and are commonly obtained from non-invasive DTI or diffusion spectrum imaging (DSI) [37], [38]. The connection matrix of the network of the human brain forms the so-called “human connectome” [35], [36], [39], [40]. Recently, Jirsa et al. [41] used connectomics to establish a framework for a “virtual brain”, in which network modeling is used to understand the intact and damaged brain. Similar to previous work, hypothetical or random deletion of nodes or edges were used to degrade the structural connectome [42]. More recently, structural and diffusion images from 14 healthy subjects were used to create spatially unbiased white matter connectivity importance maps that quantify the amount of disruption to the overall brain network that would be incurred if that region were compromised [43]. In this study, we attempt to extend the capabilities of neurocomputational models by providing a physics-based approach for predicting degraded regions of interest. Physics-based injury predictions may help inform structural connectome analysis.

In this study, neurotrauma is investigated by using finite element simulations of a single individual subjected to a simulated head impact. Tissue damage is computed using empirically based damage models that provide a link from macroscopic biomechanical deformation to mesoscopic damage. Axonal bundle tracts are explicitly modeled using a multiscale description of white matter tracts obtained from diffusion tensor imaging. Then, using the physics-based injury predictions for white matter tissue from finite element simulations, the structural brain connectivity or connectome is degraded, and various network measures are computed. This is an important contribution because finite element simulation predictions of tissue damage provide physics-based reasoning for removing nodes or degrading edges to create the “damaged” brain connection matrix. In turn, this approach may provide further insight into mild traumatic brain injury by shedding light on the relationship between mechanical stimulus to the brain and neurobiological processes that result. Furthermore, if successful, the computational framework presented herein could supplement ongoing efforts to evaluate the use of non-invasive medical imaging tools, such as diffusion tensor and spectrum imaging, to detect white matter disruption for neurotrauma diagnostics [44], [45] by providing a time-evolving history of tissue injury.

## Methods

**Figure 1:**
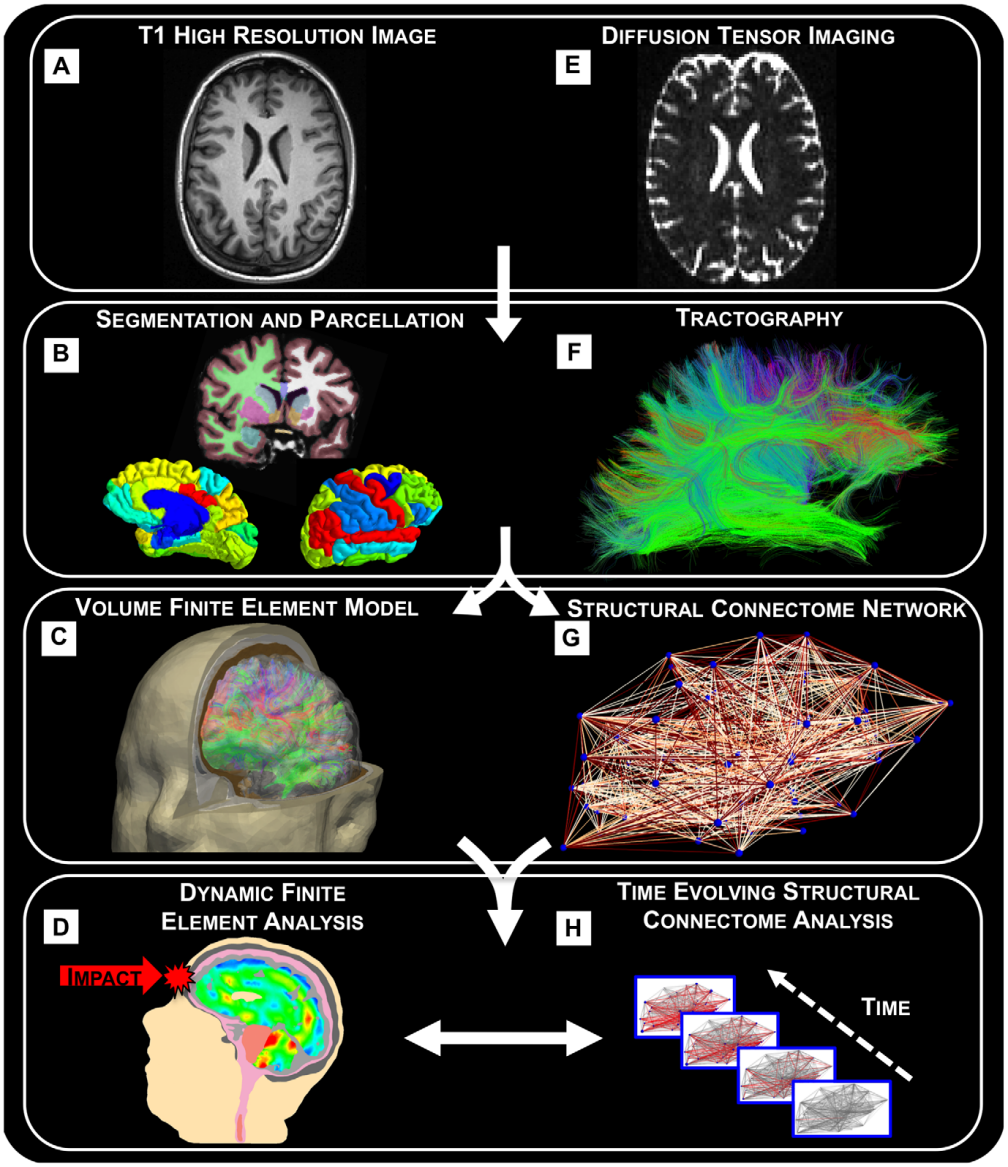
A flowchart of the process for creating the finite element model and connectome from T1 and diffusion MRI. (a) The T1 image is (b) segmented into different head materials. The segmented geometry is then used to create (c) a biofidelic three-dimensional finite element volume mesh. The mesh is required for (d) an explicit dynamic finite element simulation that captures the biomechanical response from frontal impact. (e) Diffusion tensor images are used to generate (f) axonal bundle fiber tractography which is used to inform finite element transversely isotropic constitutive descriptions of white matter tissue behavior (c). Tractography is also used to create (g) a network model of the brain that can be (h) degraded over time.

A suite of medical imaging and software tools are used to obtain an individual-specific finite element model and structural connectome-based analysis. The overarching process is schematically shown in Figure 1 and is outlined below.

### Individual Specific Models

T1 and diffusion tensor magnetic resonance images are taken from a single individual (the corresponding data can be found in [37]). The T1 image (Figure 1a) is segmented into different head materials (Figure 1b) using the software Amira [46] and the Connectome Mapper Toolkit [47]. The segmented geometry is then used to create a biofidelic three-dimensional finite element volume mesh (Figure 1c). In order to create a corresponding structural connectome or network, the T1 image is parcellated into 83 regions of interest (ROI) representing the location of anatomical regions of the brain based on the Desikan-Killiany atlas extended to include subcortical regions [48]. Since diffusion tensor images (Figure 1e) are used to generate axonal bundle fiber tractography based on the direction of peak water diffusion in each voxel [49], the DTI fiber tractography (Figure 1f) represents the approximate location of neuronal axonal bundles [50]. Fibers are filtered for connectome creation to include only fibers that begin and end within ROI. The structural connectome is assembled using the Connectome Mapper Toolkit [47] and is composed of nodes representing ROI generated at the centroid, connected by edges that represent structural pathways for which the DTI tractography traverses. Following the segmentation enabled by the Desikan-Killiany brain atlas, there are 83 network nodes and 1029 network edges. Furthermore, DTI fiber tractography is incorporated into the finite element model by using a transverse isotropic material model specifically developed for representing white matter tissue [23], [51] where each finite element within white matter regions is assigned an orientation based on the superposition of fiber tractography [52]. Details of the numerical implementation are published elsewhere [52], but it is important to point out that various different cases needed to be considered while assigning one orientation per finite element. For example, in the case of multiple fibers that overlap the spatial bounds of a single element, the multiple fiber orientations were averaged.

### Damage and Injury Thresholds for Coupling FEM Results to the Connectome Analysis

Herein, finite element simulations of the human head are designed to mimic experimental conditions for cadaveric impact tests, which are conducted to understand the dynamics of a frontal impact and the associated compression-tension damage [53]. In this study, the explicit dynamic finite element method [54] is used to capture the transient response of head impact. Supplementary information about the finite element method is included in the supporting information, Text S1. In addition to a volume mesh for the head, the finite element model also requires material constitutive descriptions and properties for all its components: skull, cortex, brain stem, cerebrospinal fluid, and soft tissue, which represents a homogenized mixture of muscle and skin. A detailed list of the material constitutive laws used to relate tissue strain to stress is included in the supporting information, Text S2. Once again, we point out that diffusion tensor imaging is used to inform the constitutive model for white matter tissue, which has been applied in the past for studying non-human primates [31] and human injury [8], [22], [55]. The entire finite element model consisted of 1,394,945 tetrahedral elements and 237,115 finite element nodes. For boundary conditions, the bottom of the neck is fixed, and a force was applied to a circular area on the forehead (about 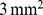) in the anteroposterior direction. The input force-time curve was a sinusoidal shape with a peak force of 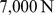 at 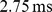. The finite element simulation computes the time-evolving mechanical strain and stress in the direction of axonal fiber bundles. The strain in the direction of axonal bundles is referred to as the axonal strain.

One limitation of the current finite element model is the exclusion of viscoelasticity in the constitutive description of brain matter. The authors acknowledge that to accurately model the progression of damage, the constitutive model should be extended to account for the time-dependent behavior of brain tissue. The exclusion may have an effect on the outcome of our results, leading to larger shear stresses, but smaller shear strains, thus, less predicted damage. For example, Chafi et al. [56] shows that viscoelasticity plays a major role in the dynamic response of the brain under blast loading. Future efforts are focused on improving the mechanical description of brain tissue.

In order to model damage using a physics-based approach, either an explicit failure mechanism should be modeled or an empirically based failure threshold is required. For this study, measures of axonal strain and strain rate computed for white matter regions are used as input for empirically based injury threshold predictions that are obtained from cellular culture experiments. Specifically, experimental results for cellular death are described using a mathematical function for tolerance criteria that relates strain to resultant cell death evaluated for up to four days post-injury [27], [32], [33]. Experimental data exists for the CA1 and CA3 regions of the hippocampus, dentate gyrus, and cortex for the rat. Experiments suggest that some regions of the rat brain are sensitive to loading rate, while other brain regions are not. That is, Elkin et al. [33] found that cortical cell death was dependent on applied strain rate, whereas hippocampal cell death was not. The relations used in the present model, obtained from Morrison III et al. [27], are:


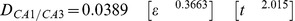
(1)


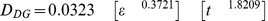
(2)


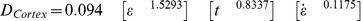
(3)

where 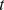 is time from insult, 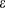 is the local strain, and 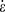 is the local strain rate [27]. Similar to Morrison III et al. [27], the units of time are days, strain is dimensionless and strain rate is inverse seconds. The damage parameter, D, is defined as the percent area of cell death. Using Equations 1–3, the axonal strain and strain rate from the finite element simulations, as well as time, are used to calculate percent cell death for a tissue region. Due to insufficient resolution of the MRI data used in this study, segmentation of the hippocampus into CA1, CA2 and dentate gyrus regions was not possible, thus the more conservative equation for 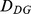 was used to compute cell death for the entire hippocampus. Equations 1–3 are monotonic functions that only increase with time, thus cellular repair mechanisms and regeneration are not currently captured. Potential issues with monotonic cellular death predictions, as well as using rat brain injury thresholds instead of human cellular injury thresholds, will be discussed later. Since the explicit dynamic finite element method is used, the transient wave propagation for solid mechanics is resolved; however, this is computationally costly and limits the total time of biomechanical prediction. The explicit dynamic simulation runs to 15 ms, thus the “long-term” structural mechanics of brain swelling, relaxation, etc., are not captured in the current model, although could be adapted in the future by using quasi-static, implicit finite element solvers.

The reader should understand that we use the local tissue strain and strain rates predicted from finite element simulations of short duration, about 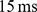, as input to experimentally based cellular death models that were developed over a 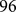 hour period. This assumes that the tissue damage due to large deformations occurs immediately and initiates an injury process that grows with time. This assumption is based on the observation that cell death was not immediate in response to deformation, but instead, increased over 4 days after injury [27].

It should be noted that the cellular death estimates that Morrison III et al. [27] developed were not based on axonal strain but instead on nominal strain applied to the back of the substrate on which the neuronal cells were attached. Thus, Equations 1–3 may not accurately describe the actual relationship between axonal strain and injury. Furthermore, the neuronal cell bodies in the experiments were not aligned in a specific orientation, so the response is not strictly representative of axonal strain injury. There are research efforts examining stretching of individual axons but have not proposed empirical relationships for cellular death in terms of applied strain, rate of loading and time from insult [57], [58]. Experimental measurements of the cellular response of white matter, especially axonal bundles, would be interesting to explore and could provide a more accurate representation of cell damage in the future.

Furthermore, the experiments performed by Morrison III et al. [27] used the rat hippocampus, which is mostly comprised of gray matter. Thus, it should be noted that the empirical data described in Equations 1–3 was not intended to describe white matter injury response so there may be limitations in applying Equations 1–3 to predict cellular white matter injury. Cellular injury threshold data for isolated white matter is currently limited; however there have been some efforts to characterize mechanical damage to axons [57]–[59]. We hope a computational framework described here will help to motivate efforts to obtain different white matter and gray matter empirical functions, which could then be used for each of region of the brain separately. As mentioned before, the application of this approach raises the possible need for understanding properties of white matter fiber bundles.

In order to map the finite element results to network-based analysis tools, output data from each finite element that represents white matter is mapped to a corresponding voxel in the MRI data that is used to create the DTI tractography. This mapping is referred to as the element-to-voxel map. Multiple finite elements within a single voxel are averaged. The element-to-voxel map enables voxels to be assigned additional data, including axonal strain and strain rate from the finite element simulation. Alstott et al. [42] chose to generate brain lesions by altering the structural connectivity matrix of the brain by deleting nodes using various methods. In the present work, instead of deleting nodes, we degrade the edges of the network based on the computed cellular death at the voxel level.

**Figure 2:**
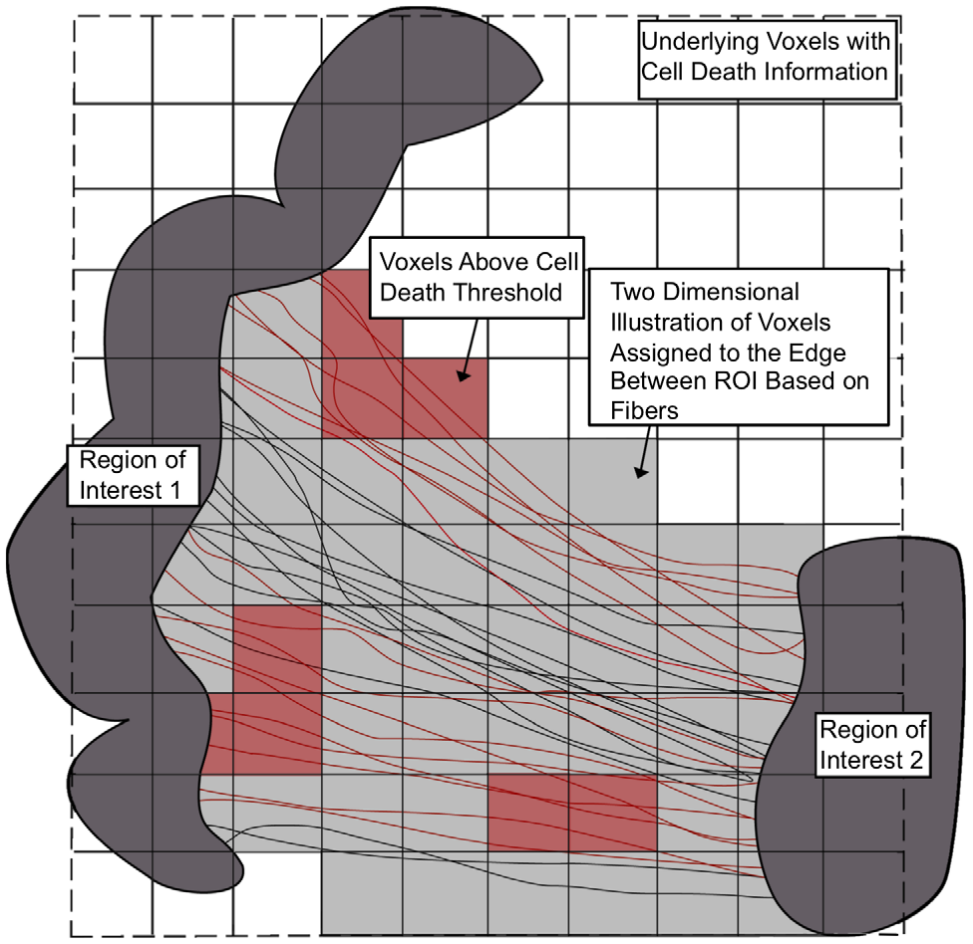
Schematic showing how structural network edges are degraded over time. Red voxels indicate that the chosen critical cellular death threshold, 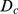, is reached. For this study, DTI tracts that traverse a damaged voxel are removed, thus degrading the connection strength of the network edge.

To understand how the structural network is degraded, consider the schematic shown in Figure 2. Edges in the network are constructed from voxels that connect two different ROIs. The edge strength is relative to the number of tracts between two ROI. Cellular death for each voxel is computed using Equations 1–3 and may grow to reach the chosen critical value of cellular death, 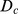 (discussed shortly). Voxels with a predicted cellular death greater than 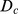 are shown in red in Figure 2. For this study, if a tract traverses a voxel that is greater than the threshold, the entire tract is considered damaged, thus the edge strength is decreased. This procedure is similar to that used by Kuceyeski et al. [43] who simulated lesions at each voxel and removed tracts passing through the lesion in order to create a damaged network to enable analysis of changes in network measures and the evaluation of the importance of each voxel. Similar to Honey et al. [60], the connection strengths were resampled to a Gaussian distribution with a mean of 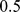 and a standard deviation of 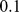. As Alstott et al. point out [42], this transformation does not alter the rank-ordering of strong to weak pathways, but simply compresses the scale of connection strengths. There are other possible ways to degrade the structural network that will be discussed later.

Since Equations 1–3 predict some degree of cell death for non-zero values of axonal strain, strain rate, and time, the additional critical value of cell death, 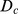, was required as a rule in order to degrade a voxel. The critical cellular death of 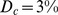 was chosen because it predicted similar levels of damage as compared to other proposed thresholds, including 18% axonal strain, which was used previously as a threshold that indicated degradation in electrophysiological function [30]. During the calculation of cellular death, positive values of strain and strain rate were used (negative strain was not used to calculate cellular death). The choice of injury threshold, as well as the micromechanics of axonal fiber bundles, is an active area of research that would assist making the current methodology more accurate in the future. During the 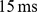 dynamic simulation, strain and strain rate data for each voxel is output at 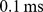 increments. Cell death is calculated at each voxel for each of these increments. Then, the maximum cell death value calculated in each voxel is used to predict cell death up to 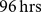. It is important to note that cell death calculations are the result of the combination of variables at each increment rather than considering each variable independently.

## Results

### Finite Element Analysis of Head Impact

**Figure 3:**
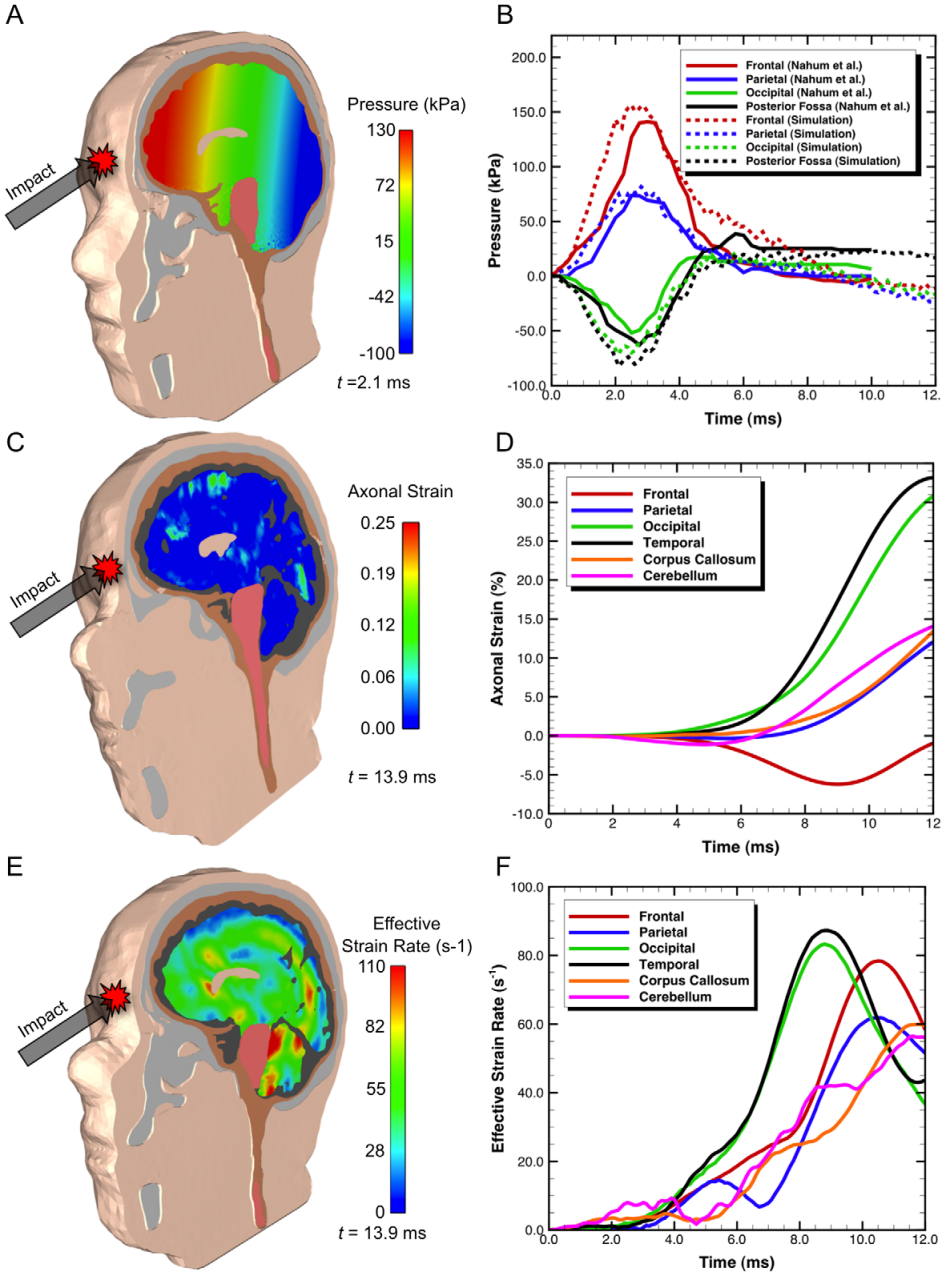
Orthographic view of the local three-dimensional response measured at various locations in the brain for (a–b) pressure, (c–d) axonal strain and (e–f) strain rate predicted using a finite element simulation for impact to the head. Prediction of the intracranial pressure response is compared to cadaveric experiments [53] and is shown in (b).

The deformed configurations of the head, along with contours and response curves for various locations within the brain (frontal, parietal, occipital, temporal, corpus callosum and cerebellum) are shown in Figures 3a–f. Output variables including pressure, axonal strain, and effective strain rate, useful for understanding the anisotropic biomechanical response for white matter, are shown. Confidence in the finite element model is established by comparing output from the computations to pre-existing experimental data on cadaveric head impact [53]. The values of axonal strain and strain rate were taken from specific locations in each of the six regions plotted in Figure 3. Four out of six of the locations represented the locations of the pressure transducers for the experiments of Nahum et al. [53]. Locations within the cerebellum and corpus callosum were also added for the strain and strain rate analysis. A variation of results will exist in each region, and taking an average of values in a anatomical region could help resolve this problem, but including simulation data points that are located farther from the experimental sampling points could also make the results less accurate. The head impact simulation took about 30 hours on 32 processors in order to reach 15 ms.

As seen in Figure 3b, the intracranial pressure quickly increases to positive values in the frontal and parietal regions, while quickly increasing to negative pressures in the occipital and posterior fossa regions. The anteroposterior pressure gradient (seen in Figure 3a) is commonly observed in experimental and computational studies - giving rise to the so-called coup and contrecoup loading scenario, for which there are an associated number of proposed injury mechanisms. Short duration intracranial pressure gradients with high positive pressures are observed at the coup region, with negative pressures at the contrecoup region. The maximum positive pressure of approximately 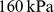 is reached in about 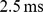 in the frontal lobe, closest to the impact, while the maximum negative pressure of approximately 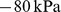 is reached in about 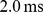 in the posterior fossa region. The computed pressure response is directly compared to Nahum et al.'s [53] experimental results in Figure 3b and show similar trends. In addition, validation of the strain response against cadaveric experiments of Hardy et al. [61] is also described in the Supplemental Text S3.

Axonal strain at the various brain regions responds slower than the pressure response. The axonal strain begins to substantially grow at 1 ms and shows a gradual rate of change of strain, with a maximum of 33% at 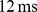 over the duration of the simulation within the temporal region. Unlike the pressure, an obvious transcranial gradient is not apparent for the axonal strain. While the frontal region had the highest predicted pressure, the temporal and occipital brain locations have the largest values of axonal strain in the regions specifically examined. Later, in the structural network analysis, these areas are associated with the largest amount of cellular death. Interestingly, if a threshold of injury of 18% axonal strain is chosen [8], [30], our results show the onset of injury occurs at approximately 9.1 ms within the temporal lobe, despite the intracranial pressure reaching approximately 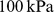 in 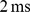. The time scales at which the pressure and strain grow will be discussed later in the context of injury cascades.

The effective strain rate, also commonly referred to as rate of loading or loading rate, has a maximum value of approximately 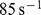 in the temporal lobe. From the contours shown in Figure 3e there appears to be a strain rate focusing, with lower strain rates closer to the skull and higher values more central to the brain. Shear strain focusing has been reported earlier [3], [62] and is attributed to the partial conversion of energy of the axial impact into a shear mechanical stress wave as a result of the material response of the brain, cerebrospinal fluid, and skull [62]. The maximum strain rate is observed before maximum axonal strain because the axonal strain accounts for magnitudes of deformation, while the strain rate relates to the rate of change of strain.

### FEM Informed Structural Connectome Analysis

**Figure 4:**
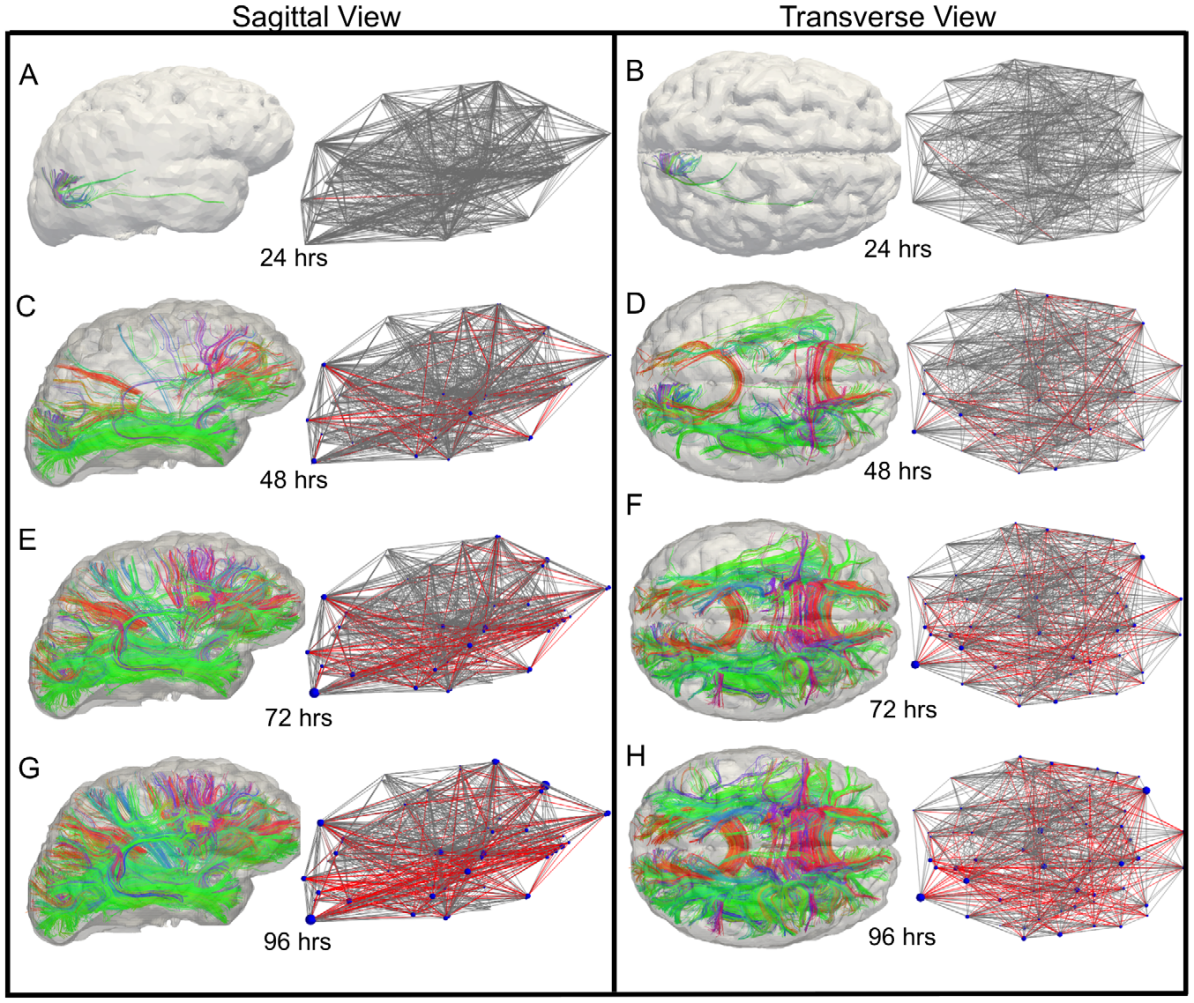
Evolution of damaged tracts and the corresponding structural networks. Using empirically-based cellular death predictions obtained from in vitro models of neural tissues, local strain and strain rate values computed from finite element simulations are used to specify injury. A computed cellular death of 3% was used as a critical value for defining white matter disruption. Damage is shown in red and the node size represents the percent change of degree. The predicted evolution of damage is shown for the sagittal and corresponding transverse views for 24 (a and b), 48 (c and d), 72 (e and f), and 96 hours (g and h).

The axonal strain and strain rate output from the finite element simulations are used to compute the amount of cellular death, according to Equations 1–3. Figure 4 shows the evolution of damaged tractography up to 96 hrs post-impact, using a critical cellular death of 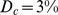 as a threshold for injury and the corresponding evolution of the degraded structural network for sagittal and transverse views. The edges in the network, which are fully damaged, are shown in red for visualization. In reality, each edge is weighted and has degraded values before it is fully damaged. The network nodes are scaled by the percentage of connections that were removed, so that larger nodes have lost more connections compared to original values (
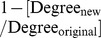
). The sagittal and transverse views are shown to provide insight about where the damage is predicted and how it progresses through time. At 24 hrs, the top four regions affected include the cuneus (medial surface of left cerebral hemisphere in the occipital lobe), fusiform gyrus (temporal lobe), lingual gyrus (occipital lobe), and peri-calcarine. Together, damage in these brain regions have 559 tractography fibers removed from network edges. Note that the total number of fiber tracts prior to impact was 497,442, so this damage corresponds to a 0.011% degradation of tractography. The percentage of fully degraded edges for 24, 48, 72, and 96 hrs are listed in Table 1. Using an 18% strain criterion, 17.3% of the edges were fully degraded.

**Table 1:** The percentage of fully degraded edges and percentage of voxels above the 3% threshold for 24, 48, 72, and 96 hrs post-injury.

Post-Injury (hrs)	% Fully Damaged	% Voxels Above
	Edges	3% Threshold
24	0.097	0.008
48	7.19	1.1
72	14.1	2.7
96	19.7	4.2

Figure 4b shows that structural damage occurs first in left cuneus and the right superiotemporal regions, resulting in one fully damaged edge at 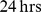. The edge between the two regions had only three tract fibers with less than one voxel or 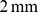 between them. The edge also had a high mean fiber length of 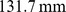 (the original total mean fiber length was 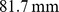). This suggests that network edges associated with few tract fibers and long fiber length are more susceptible to damage. Asymmetry of the predicted damage occurs due to the asymmetry of the underlying anatomy associated with the finite element mesh, as well as potential asymmetry of the fiber tractography, which has been studied in recent work [63]. At 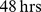, predicted damage seems to show a high density of damaged fibers in the anteroposterior direction in both hemispheres of the brain. Lateral tractography damage is also predicted within the corpus collusum. As time progresses, fully damaged rostrocaudal tracts are predicted and continue to increase.

**Figure 5:**
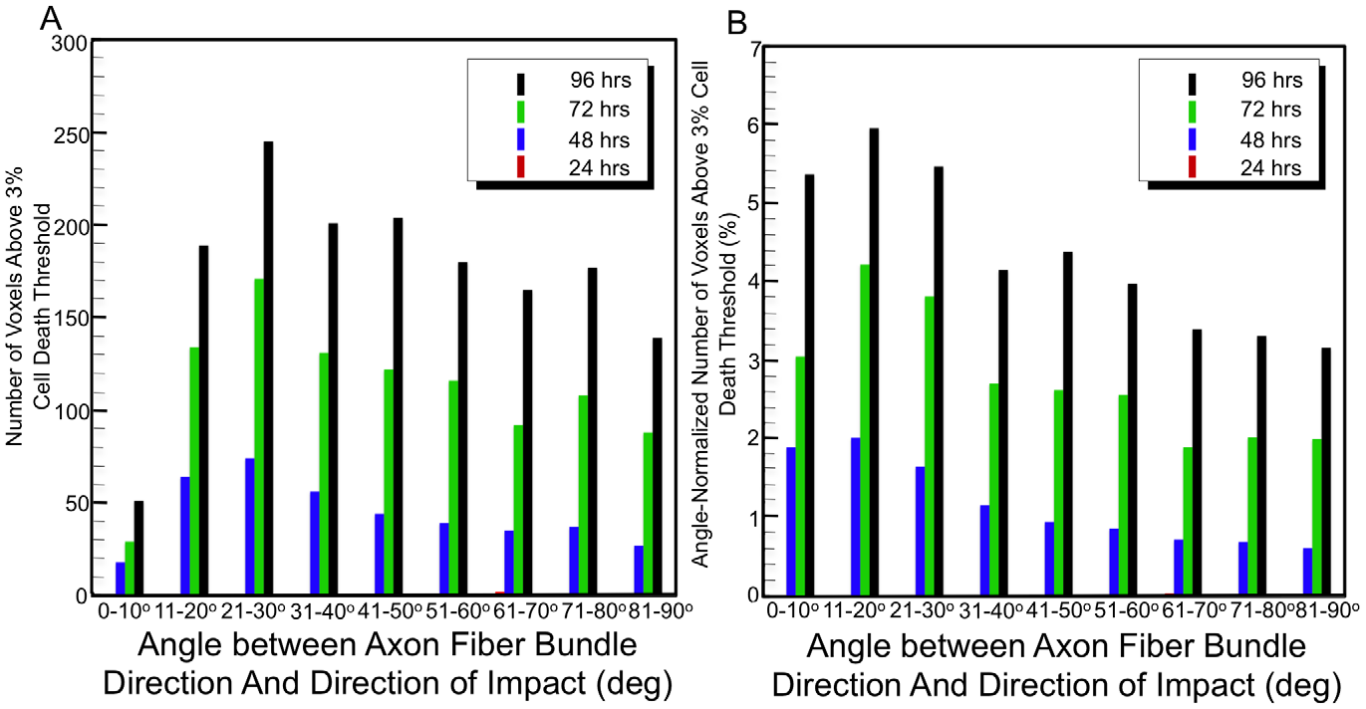
Distribution of (a) the number of voxels with predicted cellular death above 3% for each day as a function of angle between axonal fiber bundle direction and direction of impact to the head, and (b) the number of voxels with predicted cellular death above 3% for each day as a function of angle between axonal fiber bundle direction and direction of impact to the head normalized by the initial distribution of fiber angle with respect to the loading condition.

Structural changes to the tractography and resulting network arise because of the underlying voxel condition. That is, if a tract goes through a voxel that has reached the critical cellular death value, 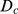, the tract is removed. Therefore, it is useful to examine how the inherent voxel properties influence results. Figure 5a shows the distribution of voxels above the predicted critical cellular death of 3% as a function of angle between the axonal fiber bundle direction and the direction of the head impact (i.e., frontal impact on the anteroposterior axis). If the tract fiber direction within a voxel is parallel with the impact direction, the angle is zero. This shows the distribution of damage as a function of angle with respect to the impact direction. Because of the high degree of mechanical rotation observed through the shear focusing in the temporal and occipital brain regions, tract fibers with large angles with respect to the impact direction are damaged initially. This is seen in Figure 5a at 24 hrs (see red bars in plot) and in Figure 4a–b.

This data can be further analyzed by normalizing the number of damaged voxels for a given orientation by the *total* number of undamaged voxels with a given orientation from the impact direction. This measure is referred to as the angle-normalized number of damaged voxels. The distribution of angle-normalized number of voxels is shown in Figure 5b, and is important because it takes into account the original distribution of the number of tract fibers eligible to be damaged with respect to the impact direction. For example, Figure 5a shows 18 voxels above the 3% cell death threshold at 48 hrs; thus, one might assume that axonal bundles oriented 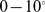 from the impact direction have little importance. However, when the original number of voxels available to be damaged is taken into consideration (Figure 5b), 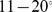 angles have the highest percentage of damage at 48 hrs. In other words, depending on the underlying white matter microstructure, i.e., the axonal bundle orientation, with respect to the direction of impact, the node, and edge degradation in the structural network may be affected differently. This is directly related to the structural mechanics (as opposed to structural connectomics) of the underlying constitutive or material law used within the finite element simulation.

However, because a monotonic function is used to describe the empirical cellular death prediction, this trend becomes more dilute as time progresses (but should not be extrapolated past 96 hrs since Equations 1–3 are not validated beyond that time). From Figure 4g–h, at 96 hours the predicted damaged axonal pathways can be seen in many directions and across all white matter brain regions indicating diffuse structural degradation. To summarize, at 24 hrs fibers in the areas of large rotational tissue strain are susceptible, while at greater times, neuronal tracts that align with the direction of impact seem more susceptible to damage (using the assumed threshold of cellular death). In the future, additional empirical cellular death predictions that are non-monotonic (if valid), or cellular regeneration and repair models, would be useful to explore.

**Figure 6:**
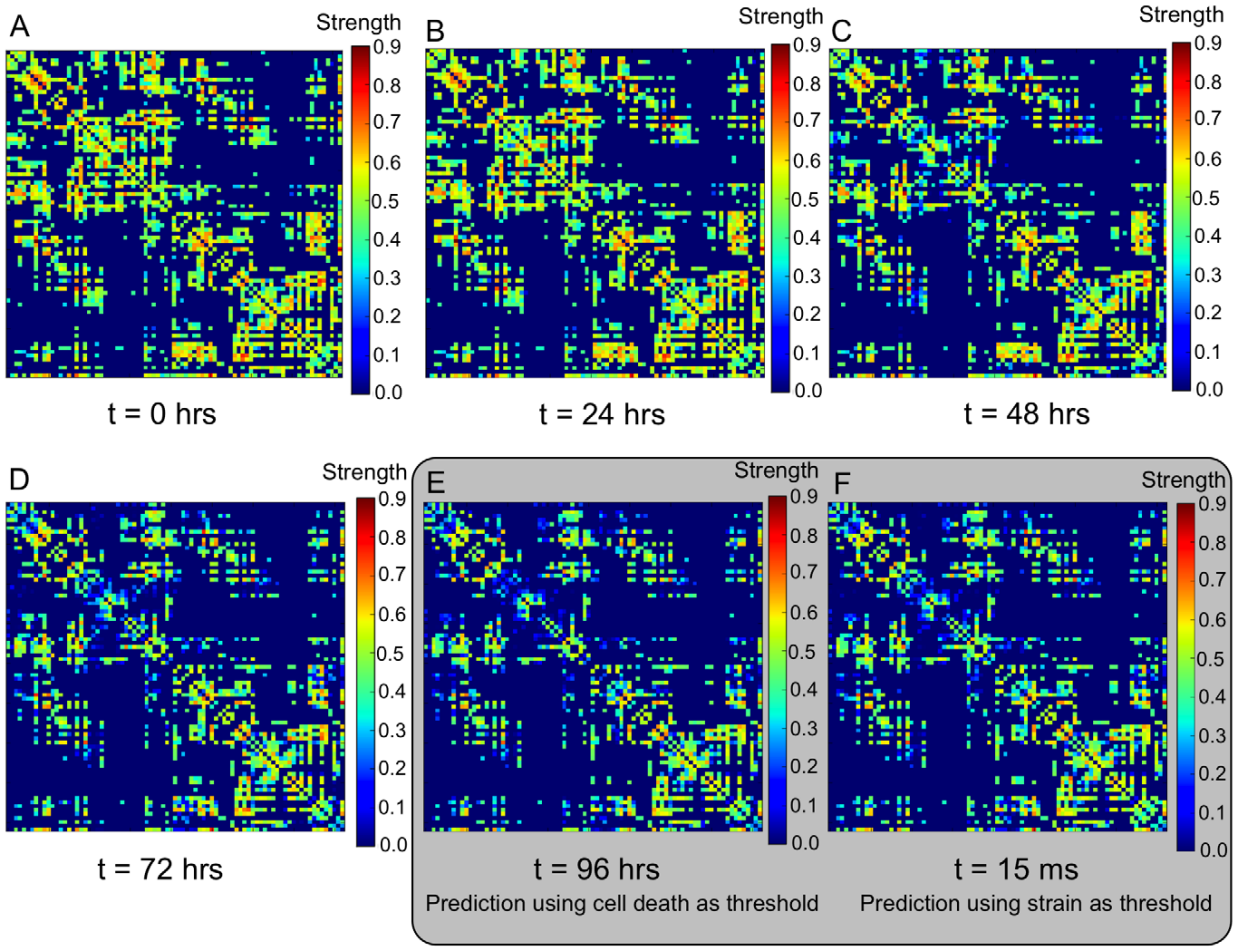
Connection strength matrices showing degradation over time, 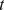. Connection strengths were resampled to a Gaussian distribution with a mean of 0.5 and a standard deviation of 0.1. Because of the monotonic cellular death criterion, as long as regions have non-zero axonal strain and strain rate from the finite element simulation, edges in the network eventually become degraded. The connection strength matrices at 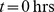 is shown in (a). The evolution of connection strength matrices for 0 (a), 24 (b), 48 (c), 72 (d), and 96 hours (e) are shown, as well as (f) the connection strength matrix for the case when a 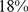 strain threshold is used.

Figure 6a–e shows the evolution of the structural connectivity strength matrices predicted as a result of head impact simulations. The original structural connectivity strength is defined as a Gaussian distribution, created by using a mean of 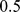 and standard deviation of 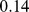 distributed over the total number of edges in the network. Each figure represents a snapshot in time starting with 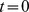 and ending at 96 hours. Within the 96 hr period (for which the monotonic cellular death criteria are validated), as long as regions have non-zero strain and strain rate computed from the finite element simulation, edges in the network become degraded. This decline in network strength is evident in Figure 6e. Since a 3% critical cellular death value is only one possible injury criteria that could be chosen, an additional criterion was examined. Results using two different damage thresholds are shown in Figure 6e and f. Figure 6e is the connection matrix using the 3% threshold at 96 hrs, while Figure 6f is connection matrix using the 18% axonal strain threshold at 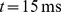, as used in previous work [8], [31]. Connection strengths show qualitatively similar trends in magnitude and location of edge degradation. While the two criteria offer a similar prediction of network damage, the cellular death Equations 1–3 are, perhaps, more useful because effects of strain, strain rate, and time have been decomposed into separate multiplicative terms that allow a compartmentalized study of extrinsic biomechanical conditions.

In general, a network's global efficiency represents how well-connected the network is compared to a perfectly connected network [64] and captures the network's capacity for communication along short paths [65]. Herein, values are reported as normalized global efficiency, which is the global efficiency of the network divided by the efficiency of an ideal network. Ideal network efficiency is calculated as a network where all nodes are connected at the minimum cost. The cost for each edge is defined as unity minus Gaussian strength. While strength offers a measure of the capacity to send information, the cost indicates the resistance to sending information - connections with high strength are low in cost. Local efficiency is a network measure that Latora [65] suggests helps to reveal the fault tolerance of the network system and shows how efficient communication between first node neighbors is. For this study, local efficiency is calculated for the same set of nodes used to make the local network of the undamaged network, even if a node loses its edge to the primary node. This method is used to provide a more direct comparison of the network after damage by accounting for edges that were removed. As a result, edges can no longer be part of a short path between nodes and cannot contribute to the efficiency, while also taking into account all nodes that should be a part of the local network without damage.

**Figure 7:**
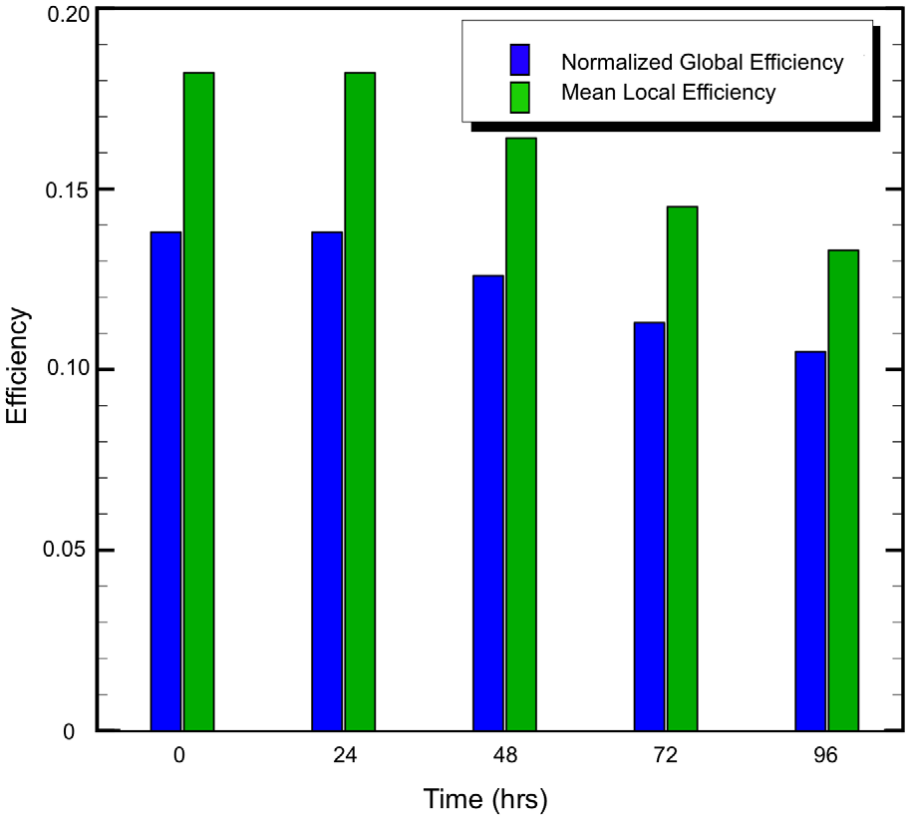
Global and local efficiency in terms of time from impact.

The normalized global and mean local efficiencies as a function of time are shown in Figure 7. The normalized global efficiency is approximately 0.14 at 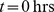 and is about the same at 24 hrs, indicating the network remains capable to send information. However, at 48 hrs an 8.8% reduction in normalized global efficiency is predicted and continues to reduce with time. A similar trend is observed in the mean local efficiency. At 96 hrs the normalized global efficiency was reduced 24%, while the mean local efficiency was reduced 27%. By 96 hrs, all but sixteen nodes had greater than 20% reduction of local efficiency. Also, note that there were no nodes completely disconnected from the network, although there were edges completely removed. The total number of edges at 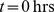 was 1029; at 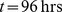, 203 edges were removed using the cellular death threshold. Using an injury threshold of 18% strain, 161 edges were removed.

Watts and Strogatz [66] define the small-world network based on the clustering coefficient of the network and the characteristic path length of the network. The clustering coefficient, 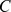, is the fraction of triangles around a node and is weighted by the geometric mean of weights associated with edges of a triangle [64]. It measures how well the first neighbors of a node are connected to each other. The characteristic path length, 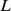, is the average shortest path between all nodes of the network. To qualify as small-world, the networks clustering coefficient should be greater than that of an equivalent random network, whereas the characteristic path length should be approximately equal [66]. The small-world coefficient is defined as 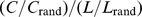 [64]. This value was found to be 1.87, 1.87, 2.03, 2.31, and 2.64 for t = 0, 24, 48, 72, and 96 hrs, respectively. When using axonal strain as the threshold variable, a value of 2.38 was computed. The increase seen in the small-world coefficient is due to the more rapid decrease in clustering coefficient of the random network compared to the damaged network that it is based on. In addition, the increase seen in the small-world coefficient also shows the ability of the network to maintain its modular structure more effectively than the random network that it was compared to.

The percent reduction of local efficiencies and the associated betweenness for the top 10 regions affected by impact at 96 hrs are listed in Table 2. Betweenness is defined as the number of times a node is part of a shortest path between nodes, and is one indication of a node's network centrality that help to describe the “importance” of a node [64], [65]. Hubs within the connectome are identified using betweenness centrality. The maximum betweenness of the original network at 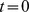 is 226, while the minimum value is zero. Recall from Figures 4a–b and 5a, white matter disruption is observed in the left cuneus and the right superiotemporal regions, which has a betweenness centrality of 41 and 17, respectively. The moderate to low betweenness of the regions first affected at 24 hrs helps to explain why the normalized global efficiency was not significantly reduced during this time and shows the importance of a robust brain network. At 96 hrs, the right hemisphere lateral orbitofrontal region shows the largest degradation in local efficiency of approximately 45.4%; however, that region also has a zero betweenness centrality. On the other hand, the right hemisphere medial orbitofrontal region has a betweenness of 110 and shows a 37.4% reduction of local efficiency, indicating it may have a more significant effect to the brain network. At 96 hrs, there are 10 network nodes with betweenness greater than 100 that have at least a 20% reduction of local efficiency. Local efficiency and betweenness for all ROIs are included in Supplemental Figures S2 and S3, respectively.

**Table 2:** Structural measures of simulated lesions for the top ten regions ranked according to percent reduction in local efficiency.

Brain Region	% Reduction	Betweenness
	Local Eff. at 96 hrs	at 0 hrs
Lateral Orbitofrontal (RH)	45.4	0
Parahippocampal (RH)	44.2	0
Parsorbitalis (RH)	44.1	0
Transverse Temporal (RH)	42.0	0
Pericalcarine (RH)	39.4	1
Parstriangularis (RH)	39.3	2
Temporal Pole (RH)	39.1	3
Rostral Anterior Cingulate (RH)	38.4	0
Frontal Pole (RH)	38.1	12
Superior Temporal (RH)	37.9	21

RH and LH refer to right and left hemispheres, respectively.

**Figure 8:**
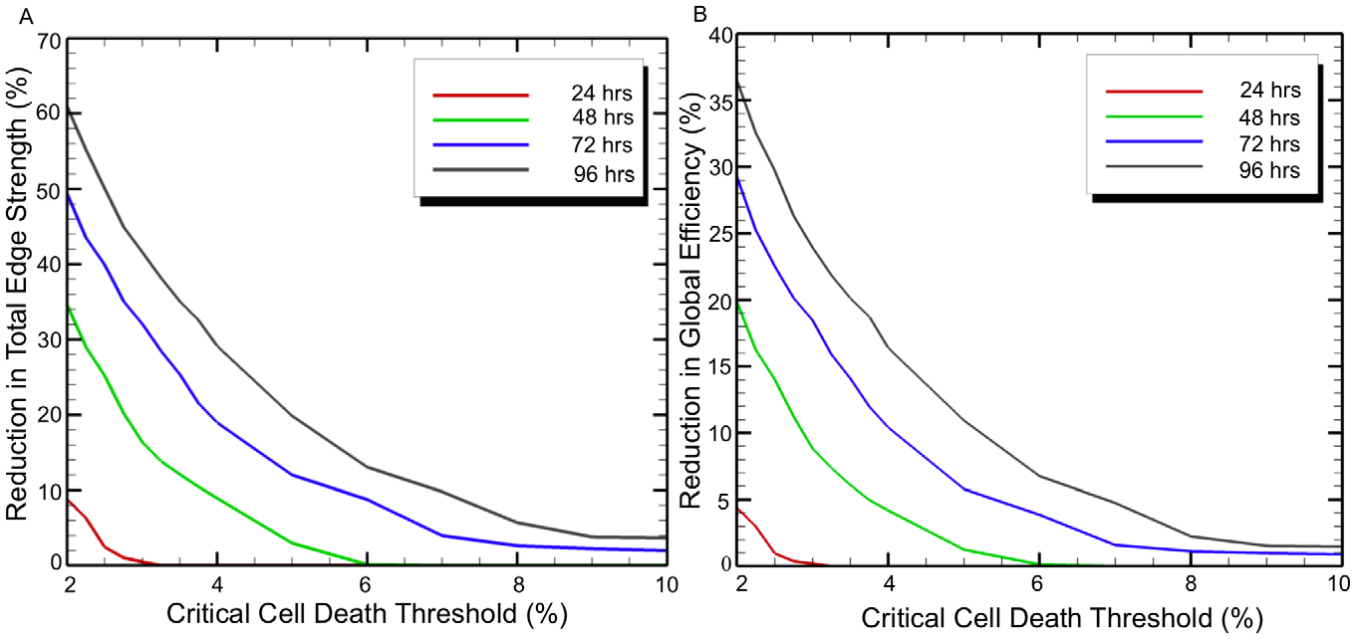
Changes in (a) total edge strength and (b) global efficiency with change in critical cell death threshold for 24, 48, 72, and 96 hours.

The results reported thus far are based on a critical cell death value of 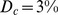, as well as a critical strain threshold of 18%. However, it is also useful to evaluate the sensitivity of the results to the choice of 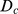. An analysis of network properties was performed for multiple critical cell death values, in the range from 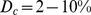, and are shown in Figure 8a–b. Figure 8a shows the percent reduction in total edge strength as a function of the critical cell death threshold choice. Figure 8b shows the percent reduction in global efficiency as a function of the critical cell death threshold choice. Results show that the choice of the critical threshold significantly affects both network measures that were examined. For example, for a change of 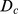 between 3% or 4%, the reduction in global efficiency is 23.9% and 16.4%, respectively. In other words, from only a 1% change in the choice of 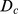 results in a 7.5% change in global efficiency reduction. Therefore, the choice of 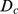 will be important in obtaining accurate results and highlights the need for future experimentation to characterize cellular injury criteria for all areas of the brain in order to improve the accuracy of this modeling approach.

## Discussion

For the first time, a physics-based model has been linked to a network-based analysis that establishes a coupled computational method to study the effects of localized structural damage or lesions. In the presented work, lesions are simulated by using a state-of-the-art finite element model of the human head, developed for a single individual directly from MRI, impacted on the forehead region. The local finite element variables are coupled to a network-based analysis through an empirically based cellular injury model. The new approach attempts to capture the spatiotemporal structural characteristics of brain trauma. Foundations of this idea stem from previous studies that attempted to establish relationships of lesion sites and the resulting functional impact [42], [67].

The first part of our study involved developing a new three-dimensional human head finite element model for simulating the biomechanical response from frontal impact and comparing it to experimental data obtained from cadavers. The finite element model is unique in that it uses diffusion tensor imaging tractography to inform structural mechanics constitutive laws of the underlying white matter anisotropy in an effort to help elucidate the injury mechanisms of impact neurotrauma. Simulations of frontal impact capture the coup and contrecoup loading profiles, including short duration intracranial pressure gradients with high positive pressures at the coup region, and negative pressures at the contrecoup region (Figure 3a–b). The computed pressure compares well to past experimental results [53]. While the frontal region had the highest predicted pressure, the temporal and occipital brain locations had the largest values of axonal strain in the regions specifically examined and responded slower than the pressure response. Because of varied mechanical tissue properties and constraints with the head, translational cranial motion causes relative brain movements that happens after peak pressure, and leads to large brain deformations and significant localized regions of axonal strain (Figure 3e–f).

Empirically based cellular death thresholds were used to predict the time-evolving damage in various brain regions based on finite element-based predictions of local axonal strain and strain rate. The biomechanical simulations predict that the temporal and occipital regions undergo the most axonal strain and strain rate at short times (less than 24 hrs), which leads to cellular death initiation that produces damage which shows dependence on angle of impact and underlying microstructure of brain tissue. The cellular death model that was used in this study is based on experimental observations that cell death was not immediate in response to deformation, but instead increased over four days after insult. Tissue damage becomes more dilute as time progresses (Figure 4g–h). At 96 hours, the predicted damaged axonal pathways can be seen in many directions and across all white matter brain regions, indicating diffuse structural degradation.

Interestingly, when using injury criteria proposed in the past, including thresholds of axonal strain [8], [30] or intracranial pressure [21], the finite element simulations predict injury within 10 ms of frontal impact. However, when using a cellular death criteria, damage takes longer to develop but offers a similar result to the resulting network strength (Figure 6). Thus, while the model may be limited in the most accurate description of cellular injury, it does seem to capture the underlying pathology of diffuse brain injury, i.e., widespread damage to axons in the brain, to some degree. While the two criteria offer similar prediction of network damage, cellular death, is perhaps, more useful because effects of strain, strain rate, and time have been decomposed into separate multiplicative terms that allow a compartmentalized study of extrinsic biomechanical conditions. On the other hand, as Morrison et al. [27] point out, purely mechanical definitions of injury thresholds may be too insensitive to identify the onset of injury for the brain, since biological tissues are alive and perform some form of active, physiological functions. For brain tissue, failure can be defined in various ways and may occur far below mechanical failure limits. Therefore, additional tolerance criterion that capture degradation of the electrophysiological function may be also required for brain tissue, since injury mechanisms that may alter neuronal function without requiring cell death are observed and may become activated more quickly than cell death [68].

This work has attempted to establish a physics-based methodology to inform structural connectome analysis. In the current model, network edges are degraded by weight rather than simply deleting nodes, in an attempt to include the effects of damage on white matter “fibers of passage” that Alstott et al. [42] refer to. It is assumed that tracks representing bundles of axons are able to be disconnected when passing through regions of high cell death, simulating reduction in the strength of a structural connection, and are related to a decrease in localized white matter integrity. All network damage was linked directly to predicted tissue deformation and predicted cellular death. Interestingly, at 96 hrs post-impact, the methods used here did not lead to any node completely disconnected from the network (although there were edges completely disconnected). At early times (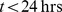) network measures of global and local efficiency were degraded little, however, as time increased to 96 hrs the network properties were significantly reduced (Figures 6 and 7). Alstott et al. [42] found that random removal of nodes did not affect network integrity until almost all of the nodes were deleted. Thus, this method may capture some structural network features of very mild neurotrauma at early times, but would benefit from a functional network analysis to explore the potential outcomes. As the network was damaged in certain areas, edges were lost or degraded, raising the cost to send information between nodes and producing different short paths between nodes. As a result, the brain network was not able to maintain efficiency by using alternate paths or finding strong hub connections past 24 hrs post-impact, thus demonstrating a potential limitation of the brain to retain network robustness in extreme conditions that cause neurotrauma.

While global efficiency is able to demonstrate widespread effects of damage, local efficiency provided a measure to investigate localized damage within the network related to areas of concentrated axonal strain and strain rate in particular areas of the brain. There was a much larger reduction in local efficiency at areas of high cell damage compared to reduction in the normalized global efficiency, indicating that the brain as a whole is resistant to some degree of localized damage. Brain regions that experienced the largest cellular death showed a larger reduction in local efficiency compared to the global efficiency of the network (Table 2). However, the low betweenness of many of these ROI suggest that they were not as necessary for communication outside their local area. This suggests that the modular nature of the network, including its small world properties, helped to prevent loss of efficiency on the global scale from damage at the local scale. This is interesting because the network considered here is a rigid, static, anatomic network in which there is no adaptability built into the model. However, it seems that damaged structural network hubs retain the ability for long distance communications. In addition, the increase predicted in the small-world coefficient also shows that modularity was not as affected by the damaged static network, as compared to a random network.

### Limitations and Future Work

There are exciting possibilities for future work, as well as limitations to the current modeling approach. The current model did not attempt to model the coupled effects to the functional network; instead, we provided an example for a single individual in order to establish a methodology to link physics-based predictions of tissue damage with structural network analysis for frontal impact neurotrauma. It is important to note that the empirical relationships may only be accurate for the rat (not the human), and most likely, there are many more regions that need to be characterized. Our results and conclusions may be altered according to these injury thresholds. Although human injury thresholds are currently limited, as additional brain region injury thresholds are experimentally characterized and improved they can be included in the future. Due to the computational cost of the finite element simulations, the brain was only segmented into 83 different regions. In the future, increased segmentation of regions of interest and improved biofidelity of the finite element model would increase the resolution of the analysis. While this study does not address the resulting functional outcome from structural degradation, coupled structure-function relationships as a result of neurotrauma would be interesting to explore. For example, a coupled analysis may enable functional stimulus that may prohibit or enhance further cell death. For the prediction of tissue damage, additional physics, such as electrochemical reactions, may be useful by incorporating diffusional properties. In addition, increased resolution of the biomechanical response may also be improved by further developing white matter material response descriptions that use multiple fiber tract orientations within a single element, thereby enabling the capability to use diffusion spectrum imaging. There is also an opportunity to use this framework to explore additional injury mechanisms or thresholds from empirical or experimental data, such as intracranial pressure. Note that the current methodology degrades network edges, instead of nodes. In the future, it may be useful to investigate methods for degrading nodes in addition to edges because the all of the nodes represent cortical gray matter, and the gray matter does experience significant strains. This would require a choice of strain measurement other than axonal strain since the gray matter is treated as isotropic. With this type of criteria, it may be possible to degrade a node based on a ratio of damaged voxels within a region of interest compared to the voxel volume of the region as a whole.

Further work should also work to validate this approach in humans. There are at least two distinct areas associated with connectome damage that should be explored in order to validate the approach described herein. The first deals with how well the location of damage within the network description is captured using physics-based predictions. The second validation strategy should address the nature in which edges and nodes in the network are degraded. There are various approaches that may be useful for addressing both areas. For example, in order to validate how well the location of damage is captured, further understanding about how cellular level changes effect fractional anisotropy, that results in altered fiber tractography would be useful to develop. Some of this information may be obtained from various ongoing studies that are examining the ability of DTI to diagnose mTBI, which could also be extended to create degraded structural connectomes. One way to do this may be to use DTI studies pre- and post-injury from typical loading conditions that cause rotation-induced diffuse axonal injury. By providing similar loading profiles within the simulation and comparing computed DTI tractography damage with the clinical data set, maybe results can be compared. Perhaps this may be accomplished using sports-related impact injury, such as American football, where many helmets have sensors built-in to record impact loads.

In conclusion, this work has explored “connectome neurotrauma mechanics” by using physics-based finite element simulations to help elucidate injury mechanisms associated with neurotrauma by using various cellular injury thresholds to define tissue damage, and established a coupled computational framework to inform structural connectome analysis.

## Supporting Information

Figure S1 The magnitude of peak relative displacement between skull and brain for validation of finite element model.
(EPS)

Figure S2 Percent reduction in local efficiency at 96 hrs for all brain regions.
(TIF)

Figure S3 Percent reduction in betweenness at 96 hrs for all brain regions.
(TIF)

Text S1 A concise description of the finite element method.
(PDF)

Text S2 Material constitutive laws and associated parameters used for the head finite element model.
(PDF)

Text S3 Additional finite element validation.
(PDF)
