# Supplementary figures and images for: Combining the Finite Element Method with Structural Connectome-based Analysis for Modeling Neurotrauma: Connectome Neurotrauma Mechanics
Source: PLoS Comput Biol. 2012 Aug 16;8(8):e1002619. doi: 10.1371/journal.pcbi.1002619 (PMC3420926; doi:10.1371/journal.pcbi.1002619)

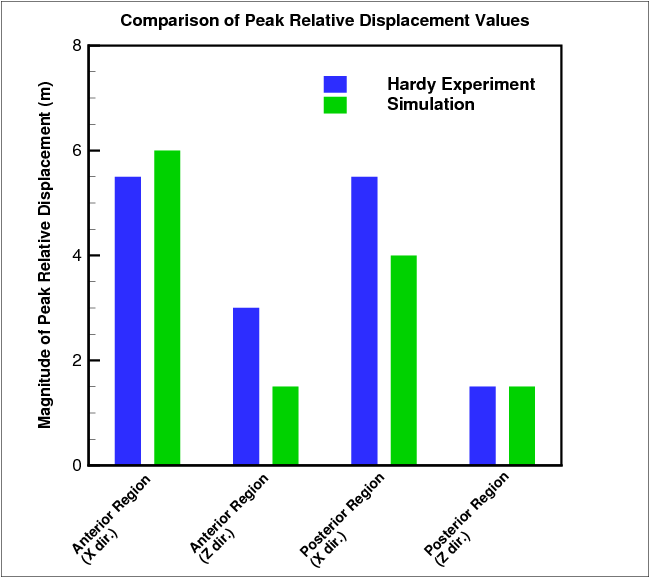

Supplement: Figure S1 — The magnitude of peak relative displacement between skull and brain for validation of finite element model. (EPS) [file pcbi.1002619.s001.tif]

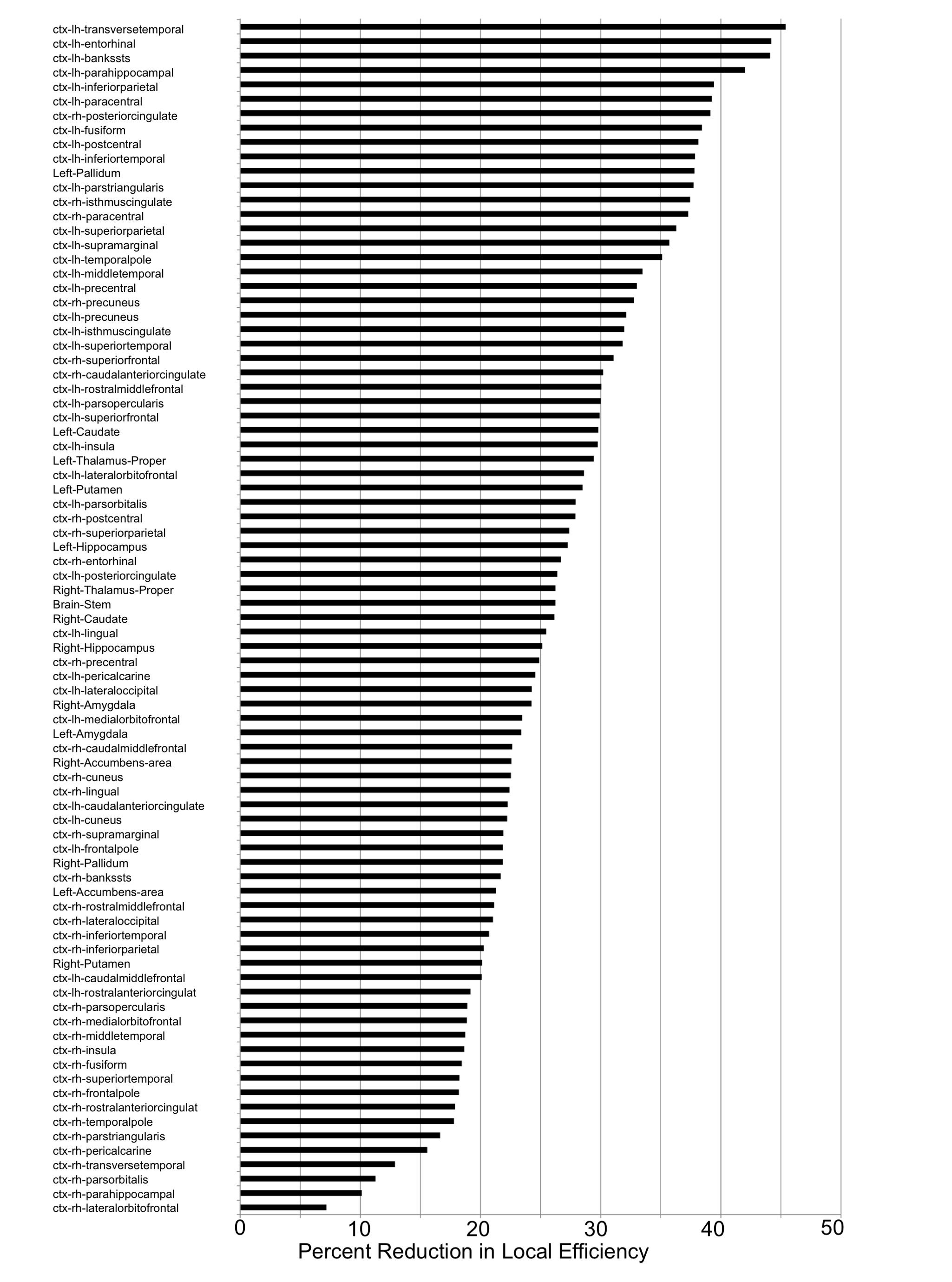

Supplement: Figure S2 — Percent reduction in local efficiency at 96 hrs for all brain regions. (TIF) [file pcbi.1002619.s002.tif]

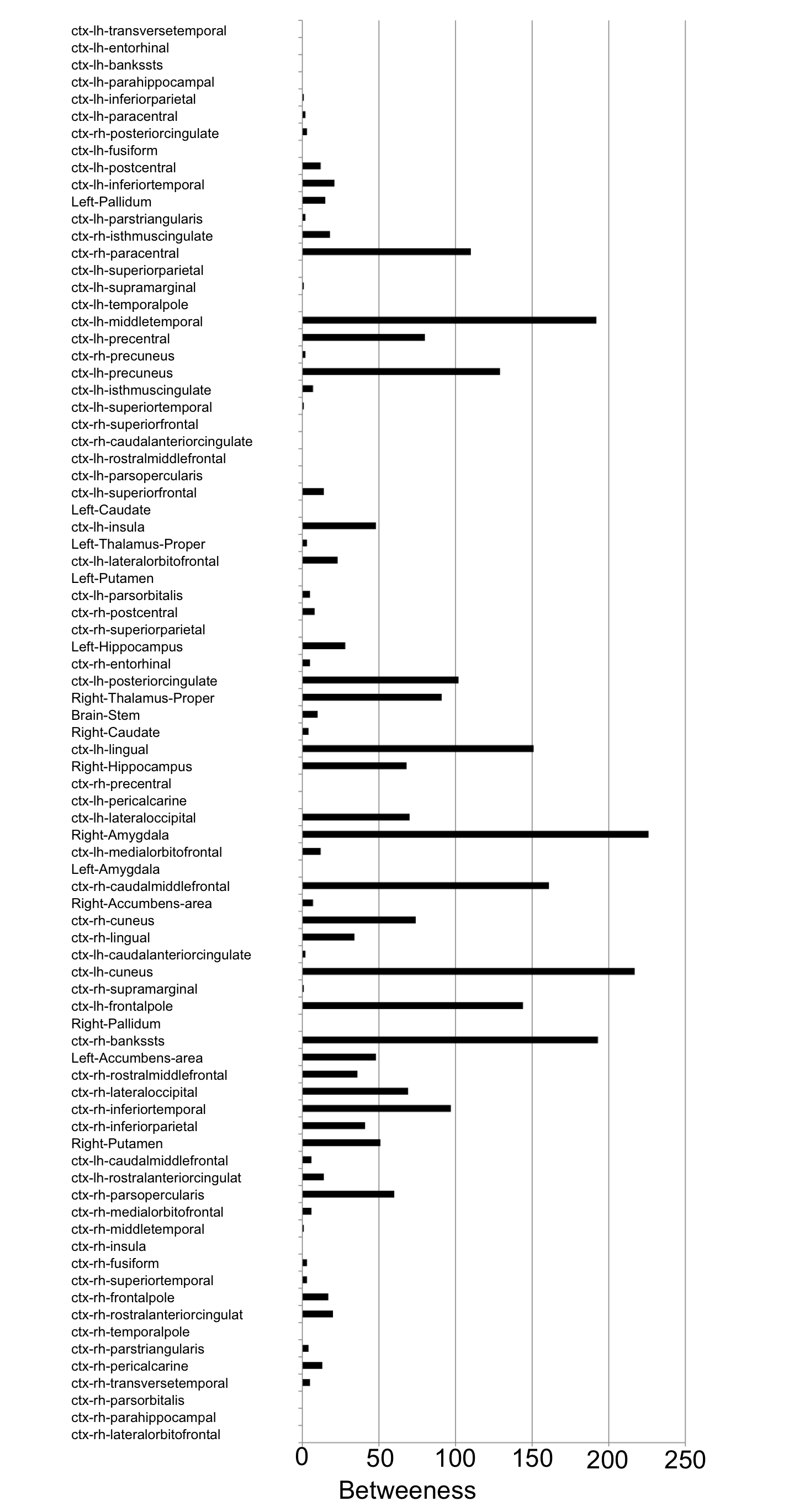

Supplement: Figure S3 — Percent reduction in betweenness at 96 hrs for all brain regions. (TIF) [file pcbi.1002619.s003.tif]
