# Supplementary material for: Combining the Finite Element Method with Structural Connectome-based Analysis for Modeling Neurotrauma: Connectome Neurotrauma Mechanics
Source: PLoS Comput Biol. 2012 Aug 16;8(8):e1002619. doi: 10.1371/journal.pcbi.1002619 (PMC3420926; doi:10.1371/journal.pcbi.1002619)
Supplement: Text S1 — A concise description of the finite element method. (PDF) [file pcbi.1002619.s004.pdf]

Supplemental Text S1

We consider the general case of a body occupying an initial configuration $B_0 \subset \mathbb{R}^3$ of the body at time t_0. Dynamic equilibrium is enforced at any time using the weak form of the virtual work principle:

$$\int_{B_0} \mathbf{P} : \nabla_0 \boldsymbol{\eta} dV_0 + \int_{B_0} \rho_0 \mathbf{b} \cdot \boldsymbol{\eta} dV_0 + \int_{\partial B_0} \bar{\mathbf{t}} \cdot \boldsymbol{\eta} dS_0 - \int_{B_0} \rho_0 \mathbf{a} \cdot \boldsymbol{\eta} dV_0 = 0 \tag{1}$$

where $\mathbf{P}$ is the first Piola-Kirchoff stress, ∇_0 denotes the material gradient, $\boldsymbol{\eta}$ is an admissible virtual displacement satisfying homogeneous boundary conditions on ∂B_0, ρ_0 is the mass density, $\mathbf{b}$ are the body forces, $\bar{\mathbf{t}}$ are the tractions applied on ∂B_0, and $\mathbf{a}$ represent the accelerations. The symbol : is used to denote the inner product between second order tensors, *e.g.*, $\mathbf{A} : \mathbf{B} \equiv A_{ij}B_{ji}$, where the summation convention on repeated indices is implied. The first term represents internal forces, the second and third terms represent external work, and the fourth term represents inertial or kinetic virtual work. Upon discretization, Equation (1) becomes:

$$\mathbf{M}\mathbf{a} + \mathbf{R}^{int}(\mathbf{x}) = \mathbf{R}^{ext}(t) \tag{2}$$

where $\mathbf{M}$ is the mass matrix, $\mathbf{a}$ is the nodal acceleration vector, $\mathbf{R}^{ext}$ and $\mathbf{R}^{int}$ are the external and internal force arrays, and $\mathbf{x}$ is the nodal coordinate array. Since we seek a temporal solution, we apply Newmark's scheme [1, 2] to determine a solution:

$$\mathbf{d}_{n+1} = \mathbf{d}_n + \Delta t \mathbf{v}_n + \Delta t^2 \left[(1/2 - \beta)\mathbf{a}_n + \beta \mathbf{a}_{n+1} \right] \tag{3}$$

$$\mathbf{v}_{n+1} = \mathbf{v}_n + \Delta t \left[(1 - \gamma)\mathbf{a}_n + \gamma \mathbf{a}_{n+1} \right] \tag{4}$$

$$\mathbf{M}\mathbf{a}_{n+1} = \mathbf{R}^{ext}_{n+1} - \mathbf{R}^{int}_{n+1} \tag{5}$$

where $\mathbf{d}$ and $\mathbf{v}$ denote the nodal displacement and material velocity fields, respectively. The parameters $\beta \in [0, \frac{1}{2}]$, and $\gamma \in [0, 1]$ control algorithm stability and accuracy. Equations (3–5) define a non-linear system of equations. Here a second-order accurate explicit version will be used. It is obtained by setting $\beta = 0$ and $\gamma = \frac{1}{2}$:

$$\mathbf{d}_{n+1} = \mathbf{d}_n + \Delta t \mathbf{v}_n + \frac{1}{2}\Delta t^2 \mathbf{a}_n \tag{6}$$

$$\mathbf{a}_{n+1} = \mathbf{M}^{-1}(\mathbf{R}^{ext}_{n+1} - \mathbf{R}^{int}_{n+1}) \tag{7}$$

$$\mathbf{v}_{n+1} = \mathbf{v}_n + \frac{1}{2}\Delta t(\mathbf{a}_{n+1} + \mathbf{a}_n) \tag{8}$$

For the explicit scheme, the mass matrix is lumped. Using this algorithm, a critical or stable time step can defined by using the longitudinal (also referred to as dilatational) wave speed, c :

$$\Delta t_{\text{stable}} \leq \alpha \left(\frac{l_e}{c} \right) \tag{9}$$

where $c = \sqrt{(\lambda + 2\mu)/\rho}$, and λ and μ are estimates of the Lamé coefficients, l_e is the minimum dimension of all elements, and α is a reduction factor to further ensure stability (chosen to be 0.8).

References

1. Belytschko T (1983) An overview of semidiscretization and time integration procedures. In: Belytschko T, Hughes T, editors, Computational Methods for Transient Analysis, Amsterdam: North-Holland. pp. 1–65.

2. Hughes TJR (1987) The finite element method: Linear Static and Dynamic Finite Element Analysis. Mineola, New York: Dover.
