# Supplementary material for: Combining the Finite Element Method with Structural Connectome-based Analysis for Modeling Neurotrauma: Connectome Neurotrauma Mechanics
Source: PLoS Comput Biol. 2012 Aug 16;8(8):e1002619. doi: 10.1371/journal.pcbi.1002619 (PMC3420926; doi:10.1371/journal.pcbi.1002619)
Supplement: Text S2 — Material constitutive laws and associated parameters used for the head finite element model. (PDF) [file pcbi.1002619.s005.pdf]

Supplemental Text S2

Material constitutive laws and associated parameters are listed in Supplemental Table SS1. Note that the white matter brain tissue is modeled using a transversely isotropic constitutive model that enables anisotropy to be included. The anisotropy is specified by using the DTI tractography to inform the the finite element of the underlying orientations of the axonal fiber bundles obtained from DTI fiber tractography. The anisotropic properties of brain tissue are important to capture within a computational framework because the axonal fiber tracts have been reported to be approximately three times stiffer than the surrounding matrix [1]. Including the axonal bundles also enables axonal strain estimates to be predicted and the associated axonal strain threshold to be specified, which provides a computational method to connect the finite element results with the network-based connectome analysis. Note that viscoelasticy is currently excluded from the constitutive response of brain tissue. This is a limitation of the current model. The inclusion of viscoelasticty may have an effect of larger shear stresses, but smaller shear strains, thus, less predicted damage. Future efforts are focused on improving the mechanical description of brain tissue.

Supplemental Table S1. Compilation of various constitutive models and parameters used for the head finite element simulation. E is the Young's Modulus, ν is Poisson's ratio, ρ is the density and K is the bulk modulus.

Anatomic Component		Material Model	Material Properties	References
Brain Tissue	White Matter	Transverse Isotropic with Mooney-Rivlin Matrix	$\rho = 1.04$ g/cm^3<br>$K = 2.3$ GPa<br>$C_{10} = C_{01} = 1.0$ kPa<br>$C_3 = 5.0$ kPa	[2–5]<br>[3,6]<br>[3]<br>[1]
	Gray Matter	Hyperelastic Mooney-Rivlin	$\rho = 1.04$ g/cm^3<br>$K = 2.3$ GPa<br>$C_{10} = C_{01} = 1.0$ kPa	[3,4,7]<br>[3,6]<br>[3]
Skull		Isotropic Elastic	$\rho = 2100$ kg/m^3<br>$E = 15.0$ GPa<br>$v = 0.229$	[3,4,8]
Cerebrospinal Fluid		Hyperelastic Mooney-Rivlin	$\rho = 1000$ kg/m^3<br>$K = 2.1$ GPa<br>$C_{10} = C_{01} = 200.0$ Pa	[3,4,9]
Skin/Muscle Layer		Elastic	$\rho = 1130$ kg/m^3<br>$E = 100.0$ kPa<br>$v = 0.45$	[3,4,9]

References

1. Arbogast KB, Margulies SS (1999) A fiber-reinforced composite model of the viscoelastic behavior of the brainstem in shear. J Biomech 32: 865-870.

2. Horgan TJ, Gilchrist MD (2003) The creation of three-dimensional nite element models for simulating head impact biomechanics. Int J Crashworthiness 8: 353–366.

3. Brands DWA, Peters GWM, Bovendeerd PHM (2004) Design and numerical implementation of a 3-d non-linear viscoelastic constitutive model for brain tissue during impact. J Biomech 37: 127–134.

4. Shafieian M, Bao J, Darvish K (2011) Mechanical properties of brain tissue in strain rates of blast injury. In: Bioengineering Conference (NEBEC), 2011 IEEE 37th Annual Northeast. pp. 1 -2. doi:10.1109/NEBC.2011.5778711.

5. Harrigan TP, Roberts J, Ward EE, Merkle AC (2010) Correlating tissue response with anatomical location of mTBI using a human head finite element model under simulated blast conditions. In: Herold K, Vossoughi J, Bentley WE, editors, 26th Southern Biomedical Engineering Conference 2010 April 30 - May 2, 2010 College Park, Maryland, USA. Springer, IFMBE Proceedings, pp. 18-21.

6. Wright RM, Ramesh KT (2011) An axonal strain injury criterion for traumatic brain injury. Biomech Model Mechanobiol : 1-16.

7. Willinger R, Kang HS, Diaw B (1999) Three-dimensional human head finite-element model validation against two experimental impacts. Ann Biomed Eng 27: 403-410.

8. Chen Y, Ostoja-Starzewski M (2010) MRI-based finite element modeling of head trauma: spherically focusing shear waves. Acta Mechanica 213: 155-167.

9. Kleiven S, Hardy WN (2002) Correlation of an FE model of the human head with local brain motion-consequences for injury prediction. Stapp Car Crash J 46: 123-144.
