# Supplementary material for: Combining the Finite Element Method with Structural Connectome-based Analysis for Modeling Neurotrauma: Connectome Neurotrauma Mechanics
Source: PLoS Comput Biol. 2012 Aug 16;8(8):e1002619. doi: 10.1371/journal.pcbi.1002619 (PMC3420926; doi:10.1371/journal.pcbi.1002619)
Supplement: Text S3 — Additional finite element validation. (PDF) [file pcbi.1002619.s006.pdf]

Supplemental Text S3

While the connectome analysis was based on the calculated head response corresponding to the experimental setup used by Nahum et al. [1], it is valuable to validate the mechanical response of finite element model against other experimental setups that used different loading conditions and measurements. For example, in the work by Hardy et al. [2], cadaver specimens were inverted and suspended in a fixture that allowed rotation and translation. The specimen underwent a frontal impact on a stationary surface with speeds that ranged between 2.5 and 3.5 m/s. In the experiments conducted by Hardy et al. [2], neutral density targets (NDTs) were implanted into 6 locations vertically arranged in the anterior and posterior regions of the brain. The three dimensional locations of the NDTs were determined using a high-speed biplanar x-ray system and the relative displacements of the brain tissue with respect to the skull was measured. Accelerative loading conditions corresponding to experiment "C383-TI" (Hardy et al. [2]) were applied to the skull of our finite element model (similar to the modeling and simulation study of Hardy and Kleiven [3]). Although relative displacements obtained from the FE model could not be sampled from the exact locations of the NDTs in the experiment, the data was sampled from six estimated locations in the anterior and posterior regions. The bar graph in Supplemental Figure S**??** compares the magnitudes of the peak relative displacement between the finite element simulation and experimental measurements. The trends qualitatively agree.

References

1. Nahum AM, Smith R, Ward CC (1977) Intracranial pressure dynamics during head impact. Proceedings of the 21st STAPP Car Crash Conference .

2. Hardy WN, Foster CD, Mason MJ, Yang KH, King AI, et al. (2001) Investigation of head injury mechanisms using neutral density technology and high-speed biplanar x-ray. Stapp Car Crash J 45: 337-368.

3. Kleiven S, Hardy WN (2002) Correlation of an FE model of the human head with local brain motion-consequences for injury prediction. Stapp Car Crash J 46: 123-144.
